# The importance of sensory integration processes for action cascading

**DOI:** 10.1038/srep09485

**Published:** 2015-03-30

**Authors:** Krutika Gohil, Ann-Kathrin Stock, Christian Beste

**Affiliations:** 1 Cognitive Neurophysiology, Department of Child and Adolescent Psychiatry, Faculty of Medicine of the TU Dresden, Germany

## Abstract

Dual tasking or action cascading is essential in everyday life and often investigated using tasks presenting stimuli in different sensory modalities. Findings obtained with multimodal tasks are often broadly generalized, but until today, it has remained unclear whether multimodal integration affects performance in action cascading or the underlying neurophysiology. To bridge this gap, we asked healthy young adults to complete a stop-change paradigm which presented different stimuli in either one or two modalities while recording behavioral and neurophysiological data. Bimodal stimulus presentation prolonged response times and affected bottom-up and top-down guided attentional processes as reflected by the P1 and N1, respectively. However, the most important effect was the modulation of response selection processes reflected by the P3 suggesting that a potentially different way of forming task goals operates during action cascading in bimodal vs. unimodal tasks. When two modalities are involved, separate task goals need to be formed while a conjoint task goal may be generated when all stimuli are presented in the same modality. On a systems level, these processes seem to be related to the modulation of activity in fronto-polar regions (BA10) as well as Broca's area (BA44).

In daily life, action control frequently requires choosing between different response options. In such situations, action control often requires the integration of different sensory modalities to achieve a goal. For example, when driving a car you may be required to stop the car in front of a red traffic light even though the navigation system instructs you to turn right immediately after this traffic light.

In cognitive neuroscience, action cascading as well as dual-tasking processes are often examined in similar situations, where responses on visual and auditory stimuli have to be carried out. A classical example for this is the psychological refractory period (PRP) paradigm which is often used to examine dual task performance. It requires two consecutive speeded responses to two stimuli presented in different modalities (e.g. visual and auditory). This typically elicits the so-called PRP-effect characterized by slower responses to the second stimulus, especially when the second stimulus is presented shortly after the first stimulus^1^,^2^,^3^,^4^,^5^. A conceptually related task making use of stimuli presented in different modalities is the Stop-Change paradigm^6^. Here, one (e.g. visual) stimulus is used to STOP an ongoing response, and another (e.g. auditory) stimulus is used to signal a CHANGE to another response alternative^6^,^7^,^8^,^9^. In this context, different results suggest that mechanisms of bottom-up and top-down attentional selection modulate performance in action cascading^9^,^10^. Even though such tasks are effective in examining ‘multi-component behavior’^11^, the obtained measures are potentially confounded by the fact that they require attentional shifting between modalities to accomplish all task goals^7^. It has been shown that attentional selection processes involved in multi-component behavior can be critically affected by the number of modalities to be processed^12^,^13^,^14^. Yet, it has remained largely elusive whether processing at the response selection level and action cascading in particular is affected by the number of sensory modalities that need to be integrated. Therefore, we aim to investigate how the process of action cascading is affected by multisensory integration.

For this purpose, we introduce two manipulations of a Stop-Change task. The first is a manipulation of sensory input modalities comparing a bimodal (visual-auditory) and a unimodal (visual) version of the task. The investigated action cascading requires stopping of a “Go” response triggered by a “STOP” stimulus, which is ultimately followed by a CHANGE stimulus. In the bimodal version, the CHANGE stimulus is presented in the auditory modality and the STOP stimulus is presented in the visual modality. In the unimodal version, STOP and CHANGE stimuli are both presented in the visual modality. Hence, both versions differ with respect to the modality of the CHANGE stimulus. This difference between task versions is expected to provide insight into the multisensory integration and attentional shifting between modalities potentially affecting action cascading. The second is a manipulation of time constraints. By presenting STOP and CHANGE stimuli either simultaneously or temporally spaced, it allows for separate vs. combined investigation of input in different modalities.

In order to investigate how bimodal vs. unimodal sensory information affects neuronal mechanisms underlying action cascading processes, we use EEG and source localization. Trying to infer how multisensory neuronal mechanisms affect action cascading processes, the P3 event related potential (ERP) is an important measure. It depicts two interrelated processes relevant to action cascading: On the one hand, the P3 reflects response selection processes during action cascading triggered by multisensory inputs^15^. On the other hand and closely related, intermodal attention shifts are also reflected by the P3^16^,^17^. Therefore, we expect both the modality manipulation and the time constraint manipulation to yield effects on response selection during CHANGE stimulus presentation. As mentioned before, the most critical aspect differentially modulating response selection in the bimodal and unimodal version might be intermodal attention shifting. Therefore, the shifting of attention between modalities to allow correct STOP and CHANGE responses should modulate the P3. Given that this is only required in the bimodal version, we expect the P3 component following the CHANGE stimulus to be smaller in the unimodal than in the bimodal version. For the same reason, the time constraint manipulation should only influence bimodal integration because temporal spacing may hinder multisensory integration^18^. Opposed to this, there should be substantially smaller response selection (P3) differences due to temporal spacing in the unimodal version because it does not require multisensory integration.

We use source localization techniques to examine how these modulations between a unimodal and a bimodal version affect the systems level. Attention shifting contributes to other executive function like cognitive branching^19^,^20^. Cognitive braching refers to the process of selecting subsequent actions based on information conveyed by past events^21^. Mechanisms of cognitive branching and attentional shifting have been suggested to be mediated by fronto-polar regions^22^. These fronto-polar regions are also modulated by multisensory integration^23^. Cognitive branching mechanisms may be more necessary in the bimodal condition, because here, information from different modalities needs to be integrated and put in order for action cascading. However, previous results suggest that the anterior cingulate cortex (ACC) plays a role in action cascading, too^7^,^10^,^24^. It is therefore possible that differences between conditions in the P3 are also related to activity changes in the ACC.

## Results

### Behavioral data

The analysis of the reaction times (RTs) on GO trials revealed no difference between the groups (F(1,30) = 1.60; p = .214; η_p_^2^ = .051). A mixed effects ANOVA using the within-subject factor “SCD interval” and the between-subject factor “group” revealed a main effect of “SCD interval” (F(1,30) = 230.56; p < .001; η_p_^2^ = .885) indicating that RTs were longer in SCD0 trials (844 ms ± 26) than in SCD300 trials (675 ms ± 28). Also, there was a main effect of “group” (F(1,30) = 19.16; p < .001; η_p_^2^ = .390) showing that RTs were generally longer in the bimodal group (878 ms ± 38) than unimodal group (640 ms ± 38). However, there was no “SCD interval x group” interaction (F(1,30) = 0.14; p > .7), which indicates that there were no differential effects of unimodal or bimodal stimulus presentation on RTs in the two SCD conditions. The SSRT did not differ between groups (p > .05).

In terms of accuracy (i.e., the absolute frequency of correct reactions), there was no group effect on GO trials (F(1,30) = 2.42; p > .13). In SC trials, the accuracy for the STOP response cannot differ because the staircase procedure was applied to assess SSRTs. Another consequence from the staircase procedure was the main effect of "SCD interval” found in the number of correct responses to the CHANGE stimulus. It showed that accuracy was higher in the SCD300 (116 ± 3.3) condition than in the SCD0 (81.3 ± 2) condition (F(1,30) = 343.90; p < .001; η_p_^2^ = .920). An interaction “SCD interval x group” was also found (F(1,30) = 10.78; p < .001; η_p_^2^ = .264), but there was no main effect “group” (F(1,30) = 12.80; p = .001; η_p_^2^ = .299). Bonferroni-corrected post-hoc independent samples t-tests were used to examine the interaction in more detail. These revealed that accuracy differed in the SCD0 condition (t_30_ = 2.55; p = .008) where it was higher in the bimodal group (85.1 ± 2) than in the unimodal group (75.5 ± 3.4). There was no difference between the groups in the SCD300 condition (t_30_ = −0.55; p > .4).

Summarizing the behavioral data, we found that the bimodal presentation of stimuli leads to a general prolonging of RTs as well as to an improvement of response accuracy in case of simultaneous inputs. Yet, a speed-accuracy trade-off can be ruled out because the RTs did not show a differential modulation across SCD conditions and groups (as was the case for the accuracy measures). The analyses reported above were repeated to control for possible age and sex effects, adding these variables as additional between-subject factor or covariate to the statistical model. All results remained the same with no effect of the additional group parameters (all F < 0.9; p > .2).

### Neurophysiological data: P1 and N1

**Figure 1:**
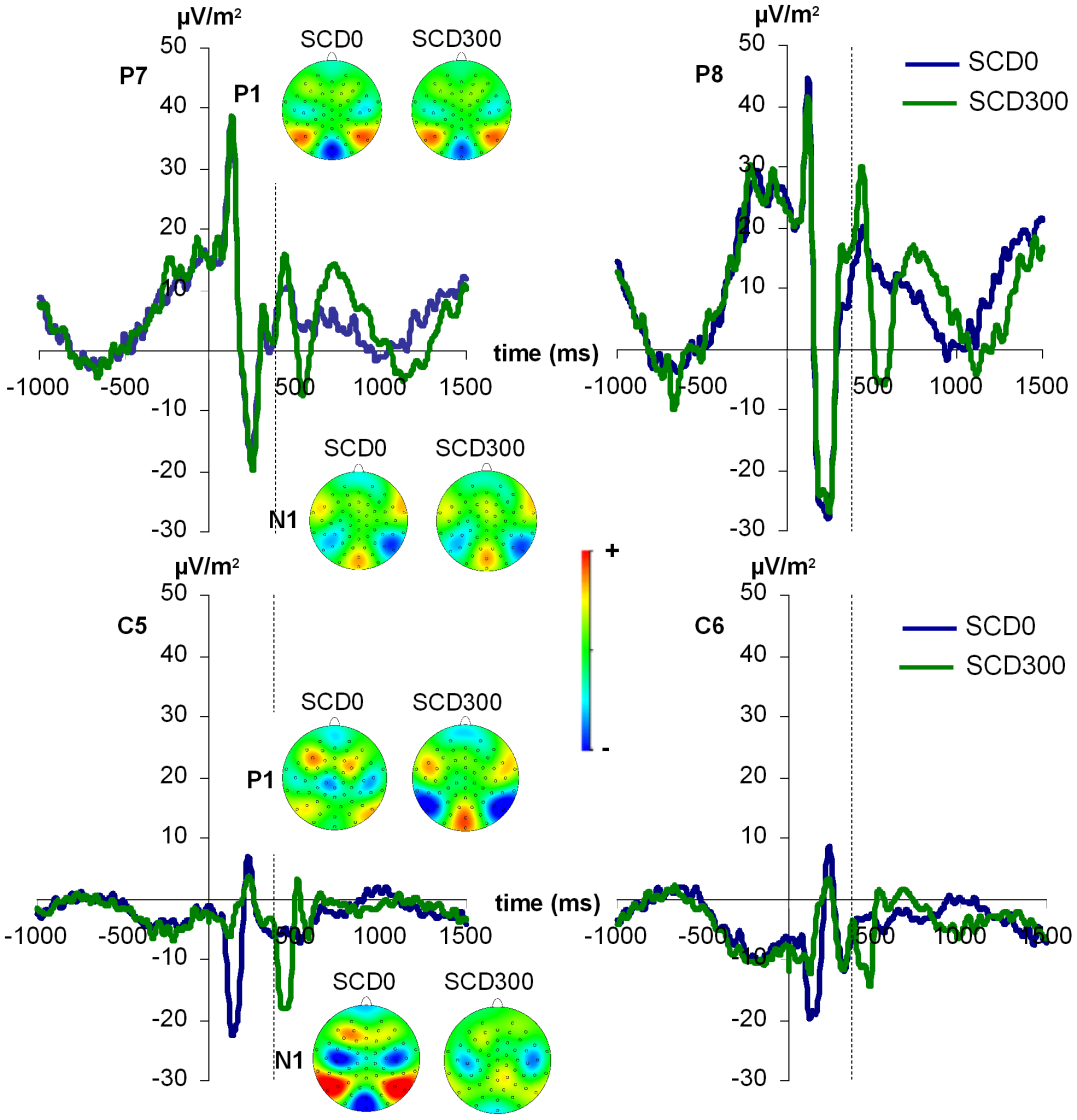
Stimulus-locked ERP curves depicting the P1 and N1 components. All curves are locked to the STOP stimulus which was presented at time point zero. In the SCD0 condition (blue lines), the CHANGE stimulus was presented simultaneously (also at time point zero), while in the SCD300 condition, the CHANGE stimulus was hence presented at time point 300. The dotted gray line at 300 ms denotes the onset on the CHANGE stimulus in the SCD300 condition. In the top row, the visual P1 and N1 elicited by the visual CHANGE stimulus in the unimodal group are displayed at electrodes P7 and P8. As can be seen from the topography maps of the peaks, electrodes P7 and P8 are located in the center of the respective positive and negative potential distributions across the scalp and thus best depict the P1 and N1 components. In the bottom row, the auditory P1 and N1 elicited by the auditory CHANGE stimulus in the bimodal group are displayed at electrodes C5 and C6. As can be seen from the topography maps of the peaks, electrodes C5 and C6 are located in the center of the respective positive and negative potential distributions across the scalp and thus best depict these components.

The ERPs on the P1 and N1 are shown in Figure 1. The P1 amplitudes were analyzed in a mixed effects ANOVA using the factors “SCD interval”, “STOP/CHANGE stimulus” (whether the ERP was elicited by a STOP or by a CHANGE stimulus) as within-subject factors and “group” as between-subject factor. The factor electrode was not modelled because any effect of electrode would be confounded by the different modalities and hence the “group” factor. The inclusion of this factor would have led to co-linearities in the ANOVA and hence to critical violations of assumptions used in ANOVA statistics.

**Figure 2:**
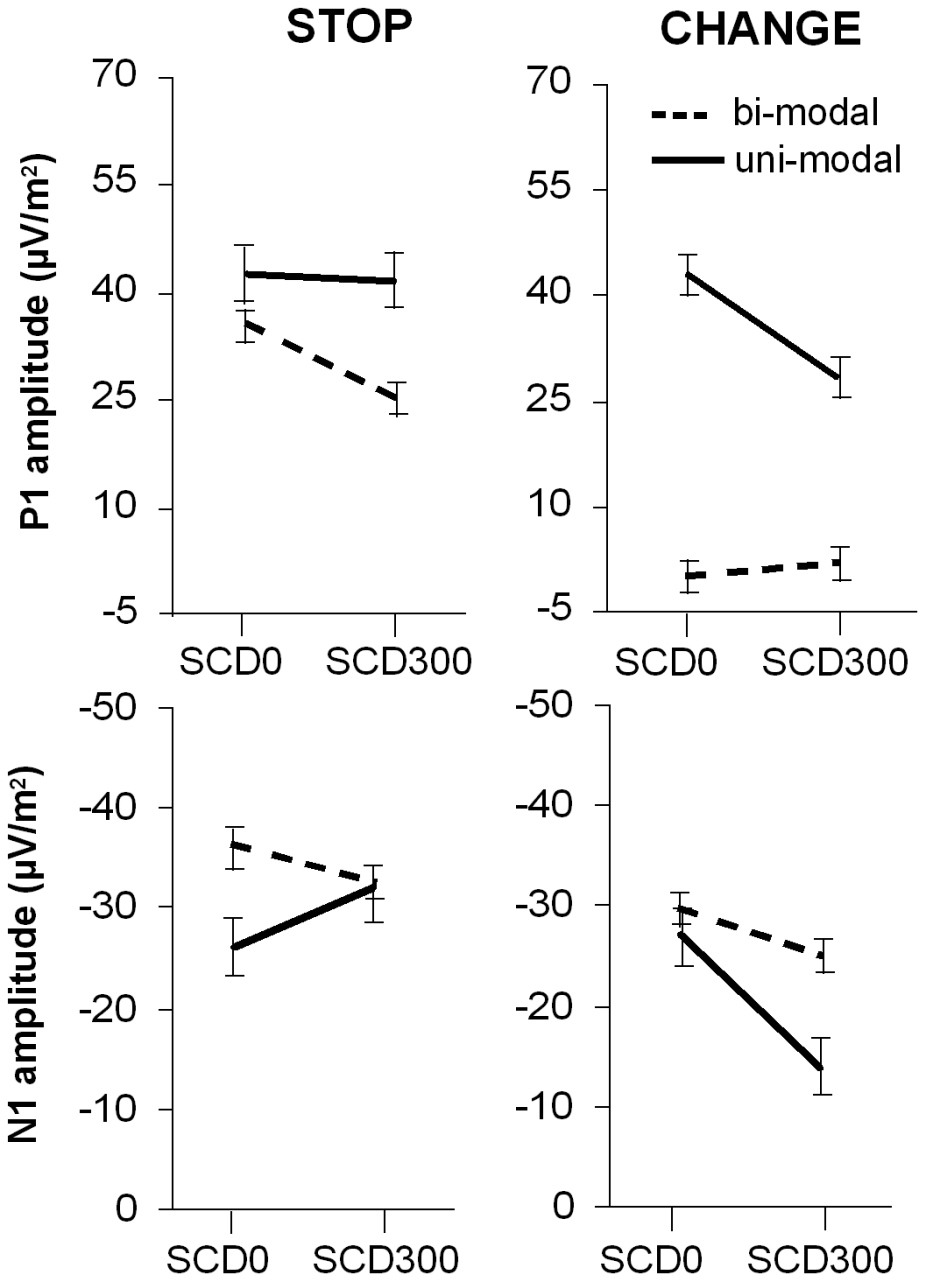
Interaction effects found for the individually quantified P1 and N1 peak amplitudes. Note that STOP stimuli were always presented visually, so that all amplitudes depicted in the left graphs were quantified at electrodes P7 and P8. In contrast, the modality of the CHANGE stimulus varied so that it was quantified at electrodes P7 and P8 for the (visual) unimodal group and at electrodes C5 and C6 fpr the bimodal (auditory) group. Differences in P1 amplitudes are shown in the top row of the graph. Most importantly, the bimodal group showed a smaller P1 amplitude in response to the visual stop stimulus when STOP and CHANGE stimuli were not presented at the same time (SCD300 condition, see top left graph). Furthermore, the auditory CHANGE stimulus presented in the bimodal task elicited equally small P1 components while the visual CHANGE stimulus presented in the unimodal task elicited a significantly larger peak amplitude when presented together with the STOP stimulus (SCD0 condition, see top right graph). Differences in the N1 amplitude are shown in the bottom row of the graph. Most importantly, the unimodal group showed a stronger decrease of the N1 amplitude in the SCD300 condition than the bimodal group (bottom right graph). In the SCD0 condition, where stimuli were presented simultaneously, the STOP signal furthermore elicited a N1 amplitude decrease in the unimodal group and an increase in the bimodal group (see bottom left graph).

The main effect of “SCD interval” (F(1,30) = 20.52; p < .001; η_p_^2^ = .406) showed that the P1 was larger in the SCD0 (30.3 μV/m^2^ ± 3.4) than in the SCD300 (23.7 μV/m^2^ ± 2.7) condition. The main effect “STOP/CHANGE stimulus” (F(1,30) = 108.59; p < .001; η_p_^2^ = .784) showed that the P1 was larger for STOP (36.4 μV/m^2^ ± 3.1) than for CHANGE stimuli (17.8 μV/m^2^ ± 3.1). However, there was an interaction of “SCD interval x STOP/CHANGE stimulus x group” (F(1,30) = 23.05; p < .001; η_p_^2^ = .435) indicating that the above main effects cannot be interpreted without accounting for the group effect. This interaction is shown in Figure 2 (top row).

Bonferroni-corrected post-hoc tests revealed that this interaction is due to the fact that within the unimodal group, the P1 was larger in the SCD0 (42.8 ± 6.6) than in the SCD300 condition (26.4 μV/m^2^ ± 5.5) for CHANGE stimuli, but not for STOP stimuli (t_15_ = 0.54; p > .3). In contrast, the bimodal group showed larger P1 in the SCD0 (35.57 μV/m^2^ ± 2.9) than in the SCD300 condition (25.63 μV/m^2^ ± 2.8) for STOP stimuli (t_15_ = 3.64; p = .001), but there were no P1 amplitude differences between the SCD conditions for CHANGE stimuli (t_15_ = −0.87; p > .2). There were no other main or interaction effects for P1 amplitudes and also no effects for P1 latency (all F < 0.8; p > .3).

For the N1 amplitudes, there was a main effect of “STOP/CHANGE stimulus” (F(1,30) = 11.59; p = .002; η_p_^2^ = .271) showing that the N1 was larger (i.e. more negative) after STOP (−31.7 μV/m^2^ ± 2.3) than after CHANGE stimuli (−24.1 μV/m^2^ ± 1.5). There was also an interaction of “SCD interval x STOP/CHANGE stimulus x group” (F(1,30) = 18.09; p < .001; η_p_^2^ = .376), which is shown in Figure 2 (bottom row). Bonferroni-corrected post-hoc tests revealed that this interaction was due to the fact that for STOP stimuli, there was a group difference (bimodal < unimodal) in the SCD0 condition (t_30_ = −1.86; p = .05) but not in the SCD300 condition (t_30_ = −0.46; p > .4). In contrast, the CHANGE stimuli showed no group differences in the SCD0 condition (t_30_ = −0.12; p > .6), but in the SCD300 condition (t_30_ = −3.63; p = .001, where bimodal < unimodal). There were no other main effets, interaction or latency difference effects evident for the N1 (all F < 1.23; p > .2).

Summing up the findings on attention-related ERP components, we found that the groups displayed differential effects. While the unimodal group showed P1 differences among the SCD conditions only after the CHANGE stimulus, the bimodal group only showed P1 differences between SCD conditions after the STOP stimulus. The direction of P1 differences was however the same in both cases (larger amplitude in SCD0 than in SCD300). The N1 showed another pattern of differential modulation. Here, the STOP-evoked N1 only differend in the SCD0 condition while the CHANGE-evoked N1 only differed in the SCD300 condition. Yet, the direction of the effect was the same (larger amplitudes in the bimodal than in the unimodal task). Controlling the analyses decrived above for possible age and sex effects revealed no effect of these additional parameters (all F < 0.4; p > .4).

### Neurophysiological data: P3

**Figure 3:**
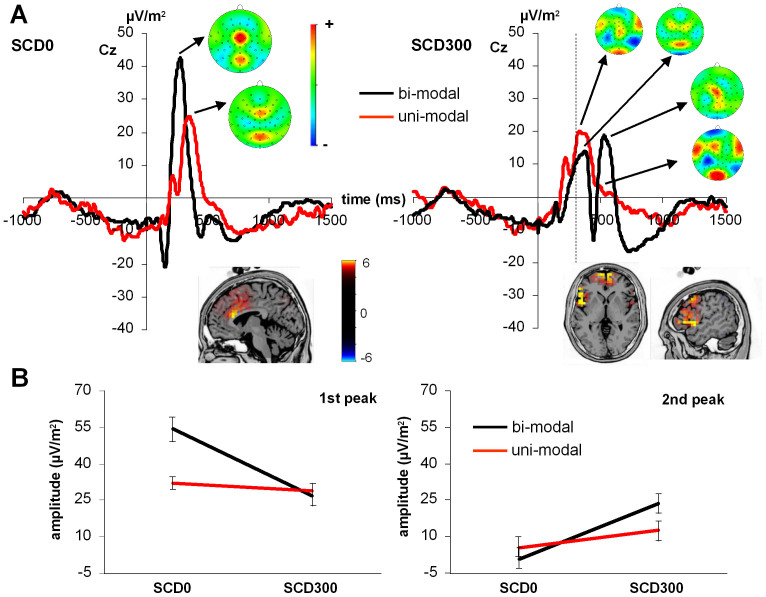
Task-dependent modulations at the level of the P3 component. (A) The top row depicts stimulus-locked ERP curves at electrode Cz. Time point zero denotes the onset of the STOP stimulus as well as the CHANGE stimulus in the SCD0 condition. The dotted gray line at 300 ms denotes the onset on the CHANGE stimulu in the SCD300 condition in the right graph. As can be seen in the top left graph, the simultaneous presentation of a visual STOP stimulus and a auditory CHANGE stimulus in the bimodal task (black curve) elicited a P3 component that was larger than the one elicited by the two visual stimuli presented in the unimodal task (red curve). sLORETA source localization revealed that the difference in peak amplitudes was due to a higher activation of the ACC (BA24) in the bimodal group. As can be seen in the topographic maps, electrode Cz best depicts the P3 component at the respective time points. The top right graph shows that the bimodal task (black line) elicited two peaks; one following the visual STOP signal and another one following the auditory CHANGE signal. By constrast, the unimodal task (red curve) only elicited a positivity following the STOP signal, but failed to elicit a second peak after the CHANGE signal. sLORETA analyses of this second peak revealed that this difference was caused by activity within the frontal pole (BA10) as well as Broca's are (BA44). (B) Interaction plots showing the modulation of the P3 peaks in the different group (unimodal vs. bimodal) across SCD conditions. The bottom left graph shows that the first P3 peak was larger in the bimodal group only when stimuli were presented simultaneously (SCD0 condition). while there was no difference. Finally, the bottom left graph shows that the peak amplitudes of increased in the SCD300 condition in both groups. Yet, the bimodal group showed a larger difference than the unimodal group.

The P3 at electrode Cz is shown in Figure 3A. In Figure 3, time point zero denotes the time point of Stop-signal presentation and the vertical dashed line denotes the presentation of the CHANGE stimulus in the SCD300 condition. In the SCD0 condition, STOP and CHANGE stimuli are both presented at time point zero. For the bimodal version two positivities can be seen around 300 and 600 ms, which confirms previous findings on this version of the task (e.g. Refs. 7, 8. In the unimodal version, however, there is no peak around 600 ms, neither in the SCD0 nor in the SCD300 condition. In this context, it may be argued that in the unimodal version, RTs were ~230 ms faster than in the bimodal version. It seems that the second peak usually observed around 600 ms in the SCD300 condition may be shifted in time and is hence reflected in the peak around 300 to 400 ms. Yet, in the unimodal version the peak is at similar latency. For the data analysis, the P3 peaks in the bimodal version were quantified as the mean amplitude in the time interval between 200 to 400 ms for the first peak and between 500 to 700 ms for the second peak. For the unimodal version, the amplitude of the potentials was quantified in the same time intervals. For the analysis of this data, an additional within-subject factor “peak interval” (first vs. second interval) was introduced in the mixed effects ANOVA.

The most complex interaction revealed by the mixed effects ANOVA was an interaction of “SCD interval x peak interval x group” (F(1,30) = 27.06; p < .001; η_p_^2^ = .474, see Figure 3B). Since this interaction involves all three factors of the statistical model, other effects are not interpretable. Post-hoc tests were performed to analyze this interaction in further detail. For the first peak interval (190 to 430 ms), the SCD0 condition elicited a significantly larger amplitude than the SCD300 condition in the bimodal group (t_15_ = 8.98; p < .001) but not in the unimodal group (t_15_ = 1.2; p > .12). For the SCD0 condition, the groups differed from each other (t_30_ = 3.19; p = .001), but there were no group differences in the SCD300 condition (t_30_ = −0.53; p > .3). A sLORETA analysis of the observed group differences in the SCD0 condition showed that differences in the first P3 peak were related to activation differences in the ACC (BA24), which was more active in the bimodal group (see Figure 3A).

For the second peak interval (450 to 740 ms), potentials were higher in the SCD300 condition than in the SCD0 condition in the unimodal group (t_15_ = −2.02; p = .031) and in the bimodal group (t_15_ = −5.99; p < .001), but the effect was stronger in the bimodal group. For the SCD0 condition, the groups did not differ from each other (t_30_ = −0.9; p > .2), but there were differences in the SCD300 condition (t_30_ = 2.52; p = .017). The SCD300 condition hence shows an inverted picture, as compared to the SCD0 condition. A subsequent sLORETA analysis of the observed group differences in the SCD300 condition showed that differences in the second P3 peak were related to activation differences in Broca's area (BA44) and the frontal pole (BA10), which were both more active in the bimodal group (see Figure 3A).

In summary, P3 peak amplitudes were differentially modulated across conditions: While the first peak only showed group differences in the SCD0 condition, the second peak only showed differences in the SCD300 condition. Event hough the direction of the amplitude differences was the same in both cases (bimodal > unimodal), different brain areas contributed to this result: In the SCD0 condition, P3 differences between the groups were due to differences in ACC activity, whereas in the SCD300 condition, Broca's area and the frontal pole were most involved in producing the bimodal P3 peak. Controlling these analyses for possible age and sex effects revealed no effect of these additional parameters (all F < 0.8; p > .2).

## Discussion

In this study, we examined how action cascading processes are differentially affected by unimodal or bimodal sensory input that needs to be processed to perform action cascading. Generally, our study shows that action cascading processes are modulated by the integration of information from different modalities.

The behavioral data show that participants were faster in the unimodal version than the bimodal task, implying that the unimodal version was easier to perform. Given that the two experiments only differ with respect to the modality in which the CHANGE stimulus is presented, the most straightforward explanation for these findings is that only in the bimodal version, attention needs to be shifted between two modalities and information from the different modalities needs to be integrated. Therefore, the detection and further processing of the reference cue (i.e. the CHANGE target) is slower when presented in a different modality than the previous visual STOP signal^25^,^26^,^27^. In line with our previous studies, we also found that due to the different temporal spacing of stimuli, participants were faster in the SCD300 than in the SCD0 condition (e.g. Refs. 7, 8. In terms of accuracy, there was neither a main group effect, nor a difference between conditions, indicating that the observed RT differences are not subject to a speed-accuracy tradeoff.

Embarking into the details of the underlying neural processes, we investigated perception and attention-related ERPs (P1 and N1) triggered by the STOP and CHANGE stimuli. The P1 is thought to provide a measure of perceptual and attentional gating and to increase with saliency of a stimulus, thus reflecting a rather automatic, bottom-up guided allocation of attentional ressources^28^,^29^,^30^. In general, P1 amplitudes were larger in the SCD0 condition than in the SCD300 condition, which may have been caused by the more complex simultaneous input in the SCD0 condition related to STOP and CHANGE stimuli. The temporal delay differentially modulated the P1. While the difference was only following the CHANGE signal in the unimodal group, only the STOP-triggered P1 was affected in the bimodal group. These findings suggest that the manipulation of input modalities had an influence on how attention was initially allocated to the stimuli. In the unimodal group, the CHANGE stimulus seemed to receive less attention in the SCD300 condition. A possible explanation for this is that it followed a visual (STOP) stimulus, thus being the second visual input and thus eliciting less bottom-up attention even though it carried relevant information. The fact that this was not found in the bimodal group can rather easily be explained by the fact that in both conditions (SCD0 and SCD300), the auditory CHANGE stimulus was equally salient because it was presented in another modality. With a task-dependent “preference” for the auditory CHANGE signal, the participants may have paid less attention to the STOP signal when presented on its own, as reflected by the smaller P1 amplitude in the SCD300 condition.

By comparison, the N1 component is thought to reflect a top-down guided discrimination process which selectively allocates attention to relevant stimulus features (e.g. Refs. 31,32,33. Here, we found that in case of simultaneous input (SCD0 condition), groups differ with respect to the STOP signal, which seems to receive more top-down guided attention in case of the bimodal task. In case of temporal spacing (SCD300 condition), this difference was however found only for the CHANGE stimulus. The finding that the bimodal group had larger N1 amplitudes in both cases can be attributed to a voluntary increase in attentional processing of stimuli in order to put up with the increased processing requirements of multimodal sensory integration.

The P3 likely reflects the process mediating between stimulus evaluation and response execution, thus depicting aspects of response selection^34^,^35^. Matching previous findings on the bimodal version of the action cascading task^15^, we found that in the bimodal task version, the P3 was locked to the CHANGE stimulus. Previous results show that modulations of the P3 mainly drive the behavioral effects between subjects using a more efficient and a less efficient strategy to cascade actions and variations in P3 amplitudes are likely due to activation differences in the ACC (BA32). When comparing unimodal vs. bimodal versions in the SCD0 condition, the sLORETA analysis suggests that the larger P3 elicited in the bimodal SCD0 condition seems to be due to greater ACC activity compared to the unimodal version. Given that ACC activity can be seen as an indicator of overall effort/processing demands (e.g. Refs. 36, 37, the larger P3 in the bimodal version in the SCD0 condition most likely reflects the increased effort or processing demand required by the multisensory integration in the bimodal task.

**Figure 4:**
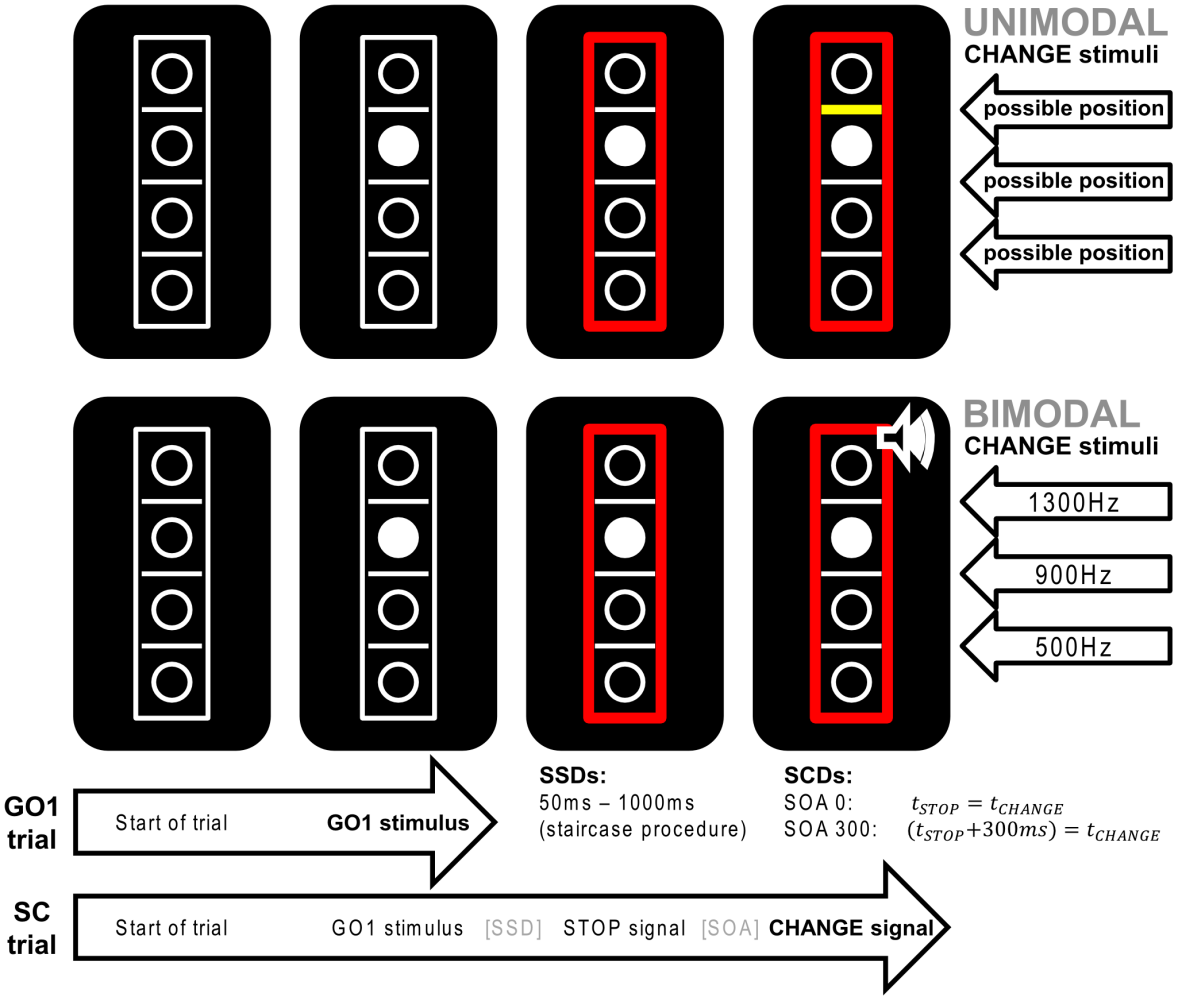
Illustration of the two versions of the stop-change task (SCT). As can be seen on the left side, both experiments presented a visual GO signal (white circle) at the beginning of all trials. In GO trials, the subjects needed to respond with the right hand (middle finger = “above” response, index finger = “below” response). In stop-change trials, the GO stimulus was followed by a visual STOP stimulus (red rectangle, see middle) after a variable and individually adjusted stop-signal delay (SSD). The CHANGE stimulus was either presented with a stimulus onset asynchrony (SOA)/stop-signal delay (SCD) of 0 ms or of 300 ms after the STOP stimulus. Our experimental manipulation was limited to the modality in which the CHANGE stimulus was presented: In the unimodal task (see top row), the CHANGE stimulus was a bold yellow line. By constrast, the bimodal task (bottom row) used 200 ms sine tones (1300 Hz, 900 Hz, and 500 Hz) as CHANGE stimuli. Responses to the CHANGE stimulus had to be given with the left hand (middle finger = “above” response, index finger = “below” response).

However, the most important finding of this study is the dissociation of the processes eliciting the P3. While in the bimodal version, the P3 always followed the CHANGE stimulus, it was bound to the STOP stimulus in the unimodal version (as indicated by the lack of a P3 peak after the CHANGE in the SCD300 condition, see Fig. 4A). In general, action cascading is achieved by means of task goal processing and manipulation^6^,^38^,^39^,^40^,^41^. For the bimodal version of the employed paradigm, Verbruggen et al. demonstrated the existence of three task goals: a GO goal, a STOP goal, and a CHANGE goal. In previous studies, we were able to show that the P3 component was related to performance in the task^15^. In the bimodal version, the P3 may be seen as an indicator of the task goal processing as well as reflecting aspects of multisensory integration. The fact that the CHANGE stimulus fails to elicit such a P3 component in the unimodal version in SCD300 condition suggests underlying differences in task goal processing or multisensory integration. A potentially different way of forming task goals might provide an explanation for our findings when assuming that task goals can be combined/merged when information stems from the same modality^42^,^43^: In the bimodal version, participants formed a STOP task goal based on visual information and a separate task goal based on the information of the auditory CHANGE stimulus. By contrast, it is possible that participants performing the unimodal version might have already begun to form a conjoint response task goal upon the presentation of the visual STOP signal given that the CHANGE signal would later occur in the same modality.

At a systems level, the sLORETA results suggest that processing differences between task versions in the SCD300 condition are related to the the frontal pole and Broca's area. These areas were more active in the bimodal task version (compare Fig. 4A). The frontal pole has been demonstrated to be modulated by multisensory integration^23^ as well as cognitive branching and task switching processes^44^. Based on our findings, this suggests that the CHANGE-locked P3 component found in the bimodal SCD300 condition reflects the integration of different sensory inputs from multiple modalities for the purpose of goal-directed action cascading. Activation differences in fronto-polar regions may be interpreted such that in the bimodal version, a switch between modalities is required (i.e., STOP stimulus in visual modality and CHANGE stimulus in auditory modality). In the bimodal version, participants had to switch from the visual to the auditory modality to respond correctly. This cross-modal switching might have delayed the P3 latency until after the CHANGE signal in the bimodal version. By comparison, the unimodal version does not necessitate such a switch across modality-specific task goals, which might provide a reason for the much earlier onset of the P3 component. Matching this interpretation, the P3 has been found to be modulated by task switches (e.g. Refs. 34, 45 and also the fronto-polar region has been shown to be involved attention shifts and hence the processes closely related to task switching between modalities^46^,^47^. Thus, the higher fronto-polar activation during the bimodal version suggests that participants performed intermodal attention shifts to respond correctly. Related to aspects of shifting, the fronto-polar activation differences might also reflect that in the bimodal version, participants had to maintain information about the running task in a pending state (i.e. maintaining the relevant information conveyed by the auditory reference cue) while performing subsequent processes (i.e. Planning and executing CHANGE response using motor and visual modalities). This is known as cognitive branching, which denotes “a process requiring holding one goal in mind while performing sub-goal processes”^48^,^49^. However, as mentioned above, the results from source localization also suggest Broca's area to stronger activated in the SCD300 in the bimodal task version. On the grounds that Broca's area is explicitly involved in the processing of hierarchical action sequences^50^,^51^,^52^, this furthermore supports the above-mentioned hypothesis that only in the bimodal task, participants maintained two separate STOP and CHANGE task goals which needed to be organized in a hierarchical fashion.

A limitation of the study is that the reported effects are based on a between-subject manipulation of a stop-change paradigm, with one group experiencing a unimodal change stimulus, while bimodal processing was required from the other group. This necessarily confounds any modality effects with group differences. The results may therefore be different when testing in a within-subject design. However, repeating the paradigm might result in a distortion of behavioral and neurophysiological parameters due to learning effects. Furthermore, the results are the same when controlling for the effects of age and sex.

In summary, we investigated if and how multisensory integration modulates action cascading processes. The data show that action cascading processes are differentially affected by unimodal or bimodal sensory input that needs to be integrated to allow multicomponent behavior. These results suggest that the manipulation of input modalities influenced how attention was allocated to the different stimuli. Yet, the modulation of response selection processes, i.e. the dissociation of processes eliciting the P3 in the bimodal and unimodal task versions, was most important. While the P3 was strongly modulated by the CHANGE process in the bimodal version, it was strongly modulated by the STOP process in the unimodal version. A potentially different way of forming task goals might provide an explanation for these findings. When two modalities are involved, separate task goals need to be set up whereas a conjoint task goal may be set up when all stimuli are presented in the same modality. On a systems level, these processes seem to related to modulations of activity in fronto-polar regions (BA10) as well as Broca's area (BA44).

## Methods

### Sample

Our sample consisted of n = 32 healthy right handed participants (18 females) aged 19–30 (mean age = 24.65 ± 2.92). All of the participants stated to be right-handed and to have no history of psychiatric or neurologic diseases. Each participant was randomly assigned to one of two experiments (visual or auditory version of the task; n = 16 auditory and n = 16 visual experiments). All participants had normal hearing abilities and normal or correct-to-normal vision. All participants were naïve to the experimental design. Each participant gave written informed consent before beginning the experiment. After the experiment, each of them was reimbursed with 10 [euro]. The study and all experimental protocols were approved by the ethics committee of the Faculty of Medicine of the TU Dresden and was carried out in accordance with the Declaration of Helsinki. Demographical data are shown in Table 1. This table also includes information about the number of rejected trials during EEG data processing. There were generally no differences in these parameters between the experiments (all p > .4).

### General experimental paradigm

All subjects were comfortably seated at a distance of 57 cm from a 17 inch CRT computer monitor in a sound-attenuated room. The participants were instructed to respond using four different keys (“S”, “C”, “N”, and “K”) located on a regular computer keyboard placed in front of them. “Presentation” software (Version 17.1 by Neurobehavioral Systems, Inc.) was used to present the stimuli, record the behavioral responses (Reaction times (RTs) and correct responses) and to synchronize with the EEG.

A modified version of the Stop-Change paradigm introduced by Verbruggen et al. was used in this study (see Figure 4 for illustration). The task consisted of 864 trials and lasted for about 25 minutes. Two thirds of the trials were “GO” trials and the remaining trials were “Stop-Change” (SC) trials. Both of these trial types were presented in a pseudorandomized order. The task was presented on a black background. The task array consisted of 4 vertically arranged white bordered circles separated by 3 white horizontal lines. This task array was enclosed in a white-bordered rectangle (as shown in Fig. 1). Each trial began with this empty array and after 250 ms, one of the four circles was filled with white color. In the “GO” condition, this white circle became the target and participants were asked to respond to it by pressing one of two keys with the right hand. In case the target was located above the middle white line, participants had to respond with their right middle finger and if the target was located below the middle line, participants had to respond with their right index finger for the correct key response. If participants did not respond within 1000 ms after the onset of the target, a speed up sign (the German word “Schneller!” which translates to “Faster!”) was presented above the stimulus array until the trial was ended by a button press. SC trials also began with the empty array followed by the GO stimulus. After a variable Stop-signal delay (SSD), the GO stimulus was followed by a STOP stimulus (the border of the rectangle turned from white to red, see figure 1). The SSD was adjusted to each participant's individual task performance by means of a staircase algorithm (cf. Refs. 6, 53. The SSD was initially set to 250 ms. If the participant did not make any mistakes during a SC trial (hence did not respond before the presentation of the STOP stimulus and correctly responded to the CHANGE stimulus described below) the SSD was decreased by 50 ms. In case of any incorrect response, the SSD was increased by 50 ms. Hence, the staircase yielded a 50% probability of successfully performed SC trials. To keep the trial duration within reasonable limits, SSD variation was restricted to a range from 50 to 1000 ms.

The STOP stimulus was followed by a CHANGE stimulus requiring the participants to respond with their left hand instead. There were two SC conditions. In the first condition, there was no Stop-Change delay (SCD0) so that STOP and CHANGE stimuli were presented simultaneously. In the second SC condition, there was a stimulus onset asynchrony of 300 ms (SCD300) so that the CHANGE stimulus was presented 300 ms after the onset of the STOP stimulus. Our two experiments used visual and auditory CHANGE stimuli (see below) which, irrespective of the input modality, were indicative of one of the three lines. In each experiment, the participants were asked to spatially relate the target (white circle) to the new reference line. In case the target was located above the reference line, participants had to respond with their left middle finger and if the target was located below the reference line, then participants had to respond with their left index finger for the correct key response. In case participants did not respond within 2000 ms after the onset of the CHANGE stimulus, the speed up sign was presented above the stimulus array until the trial was ended by a button press.

### Visual and auditory experiments

Half of the participants completed a visual version of the Stop-Change paradigm. Here, the CHANGE stimuli were bold yellow bars, which remained on the screen until the participant responded by pressing one of the response keys. In each SC trial, one of the three horizontal lines would turn into a bold yellow bar, thus becoming the new reference line.

In the auditory experiment, the CHANGE stimulus was a 200 ms sine tone presented via headphones. There were 3 differently pitched tones (low/600 Hz, middle/900 Hz, and high/1200 Hz) presented at a 75 dB sound pressure level. The middle tone represented the middle reference line while the high and low tones stood for the high and low reference lines, respectively. Given that all tones were presented to the two ears via headphones, they did not have inherent spatial properties, as compared to the visual CHANGE stimuli.

### EEG Recording and Analysis

High-density EEG recording was acquired using a QuickAmp amplifier (Brain Products, Inc.) with 65 Ag–AgCl electrodes at standard scalp positions. The reference electrode was located at Fpz. The data were recorded with a sampling rate of 1000 Hz and later (offline) down-sampled to 256 Hz. All electrode impedances were set to <5 kΩ. After recording, a band- pass filter ranging from 0.5 to 20 Hz was applied and manual inspection of the data was performed to remove technical artifacts Then, in order to correct the periodically recurring artifacts such as eye blinks or saccade artifacts, an independent component analysis (ICA) was applied using the Infomax algorithm. Independent components reflecting artifacts were discarded before back-projecting the data. Afterwards, one more manual raw data inspection was applied to remove any residuall artifacts. Next, the EEG data was segmented according to the two SCD conditions (SCD0 and SCD300). The segmentation was performed in relation to the occurrence of the STOP signal^7^. After the data was epoched, an automated artifact rejection was applied. The rejection criteria included a maximum voltage of >60 μV/ms, a maximal value difference of 150 μV in a 250 ms interval, or activity <0.1 μV. In order to eliminate the reference potential, a current source density (CSD) transformation was applied^54^. The CSD also works as a spatial filter^55^, which helps to identify the electrodes that best reflect activity related to different cognitive processes. Then, a baseline correction was set to the time window from −900 till −700 ms to obtain a “real” prestimulus baseline. Based on this stimulus locking procedure, the P1, N1, and P3 ERPs were quantified. Electrodes were chosen on the basis of visual inspection of the scalp topography which it showed a bilateral pattern of activation for the different ERP components. Due to this bilateral pattern, electrodes on both sides of the scalp were quantified. Hence, the visual P1 and N1 were measured at electrodes P7 and P8 (P1: 50–160 ms and N1: 160–300 ms post-stimulus, respectively), the auditory N1 was measured at C5 and C6 (90–190 ms post-stimulus), and the P3 was measured at Cz (first peak190–430 ms and second peak 450–740 ms after the onset of the STOP stimulus). A validation procedure was run to decide the choice of these electrodes: A search interval was defined for each ERP component, in which the component was expected to be maximal. Then, the mean amplitude within each of these search intervals of each of the 65 electrode positions were extracted. This was done after CSD transformation of the data which accentuates scalp topography^55^. Following this, Bonferroni-correction for multiple comparisons (critical threshold, p = 0.0007) was used to compare each electrode against an average of all other electrodes. Only electrodes showing significantly larger mean amplitudes (i.e., negative for the N1-potentials and positive for the P-potentials) as compared to other electrodes were chosen. This procedure revealed the same electrodes as previously chosen by visual inspection of the scalp topography plots. All components were quantified in peak amplitude and latency on the single-subject level. The P1 and N1 ERP amplitudes were quantified relative to the prestimulus baseline. The P3 amplitudes were quantified in a peak-to-peak manner because the preceeding negativity was distinctively larger for the two experimental groups (refer Fig. 4A, left).

### sLORETA

ERPs source localization was conducted using sLORETA (standardized low-resolution brain electromagnetic tomography^56^,^57^. Based on extra-cranial measurements, it provides a single linear solution to the inverse problem without a localization bias^56^,^57^,^58^,^59^ and has been validated in simultaneous EEG/fMRI studies^60^. sLORETA partitions the intracerebral volume in 6,239 voxels at a spatial resolution of 5 mm. For each voxel, the standardized current density is calculated in a realistic head model^61^ using the MNI152 template. For this study, we separately compared the two experimental groups in the SCD0 and SCD300 conditions using the built-in voxel-wise randomization tests with 3,000 permutations (based on statistical nonparametric mapping). Voxels with significant differences (p < .05, corrected for multiple comparisons) between the unimodal and bimodal group were located in the MNI brain and Brodman areas (BAs). Coordinates in the MNI brain were determined using the sLORETA software (www.unizh.ch/keyinst/NewLORETA/sLORETA/sLORETA.htm). sLORETA has mathematically been proven to show reliable estimates of underlying cortical sources of ERPs^59^.

### Statistics

Mixed effects analyses of variance (ANOVAs) were used to analyze behavioral and ERP data. The factors “condition” (GO trials, SCD0 trials and SCD300 trials) and “electrode” (only for ERP data) were used as within-subject factors. The factor “group” (visual/unimodal vs. auditory/bimodal) was used as between-subjects factor. The degrees of freedom were adjusted using Greenhouse-Geisser correction. All post-hoc tests were Bonferroni-corrected. Kolmogorov–Smirnov tests indicated that all variables used for the analysis were normally distributed (all z < 0.5; P > 0.4; 1-tailed). For all descriptive statistics, the standard error of the mean (SEM) was used as a measure of variability.

## Author Contributions

C.B. conceived and designed the study. K.G. and A.K.S. carried out data collection and analysis. All authors wrote and reviewed the manuscript.

## Figures and Tables

**Table 1:** The table provides demographical data of the sample as well as the percentage of trials included in the ERP averages for each experimental condition in the different experiments (the mean and standard deviation is given in brackets)

Parameter	Visual-visual experiment	Auditory-visual experiment	Significance
Sex	9 females, 7 males	8 females, 8 males	p > .6
Age	24.2 (3)	25.1 (3.3)	p > .4
Years of education	11.4 (0.7)	11.5 (0.8)	p > .8
percentage of discarded SCD 0 trials	2.5 (0.4)	2.3 (0.6)	p > .7
Percentage of discarded SCD 300 trials	2.6 (0.5)	2.7 (0.6)	p > .7

## References

[b1] Beste C., Yildiz A., Meissner T. W. & Wolf O. T. Stress improves task processing efficiency in dual-tasks. Behav. Brain Res. 252C, 260–265 (2013). 23769959 10.1016/j.bbr.2013.06.013

[b2] Lien M.-C. & Proctor R. W. Stimulus-response compatibility and psychological refractory period effects: implications for response selection. Psychon. Bull. Rev. 9, 212–238 (2002). 12120784 10.3758/bf03196277

[b3] Pashler H. Dual-task interference in simple tasks: Data and theory. Psychol. Bull. 116, 220–244 (1994). 7972591 10.1037/0033-2909.116.2.220

[b4] Wu C. & Liu Y. Queuing network modeling of the psychological refractory period (PRP). Psychol. Rev. 115, 913–954 (2008). 18954209 10.1037/a0013123

[b5] Yildiz A. & Beste C. Parallel and serial processing in dual-tasking differentially involves mechanisms in the striatum and the lateral prefrontal cortex. Brain Struct. Funct. 10.1007/s00429-014-0847-0 (2014). 25017192

[b6] Verbruggen F., Schneider D. W. & Logan G. D. How to stop and change a response: the role of goal activation in multitasking. J. Exp. Psychol. Hum. Percept. Perform. 34, 1212–1228 (2008). 18823206 10.1037/0096-1523.34.5.1212

[b7] Mückschel M., Stock A.-K. & Beste C. Psychophysiological mechanisms of interindividual differences in goal activation modes during action cascading. Cereb. Cortex N. Y. N 1991 24, 2120–2129 (2014). 10.1093/cercor/bht066 23492952

[b8] Stock A.-K., Arning L., Epplen J. T. & Beste C. DRD1 and DRD2 Genotypes Modulate Processing Modes of Goal Activation Processes during Action Cascading. J. Neurosci. 34, 5335–5341 (2014). 24719111 10.1523/JNEUROSCI.5140-13.2014 PMC6608997

[b9] Yildiz A. *et al.* Feeling safe in the plane: Neural mechanisms underlying superior action control in airplane pilot trainees-A combined EEG/MRS study. Hum. Brain Mapp. 10.1002/hbm.22530 (2014). PMC4452896 24753040

[b10] Yildiz A., Wolf O. T. & Beste C. Stress intensifies demands on response selection during action cascading processes. Psychoneuroendocrinology 42, 178–187 (2014). 24636514 10.1016/j.psyneuen.2014.01.022

[b11] Duncan J. The multiple-demand (MD) system of the primate brain: mental programs for intelligent behaviour. Trends Cogn. Sci. 14, 172–179 (2010). 20171926 10.1016/j.tics.2010.01.004

[b12] Duncan J., Martens S. & Ward R. Restricted attentional capacity within but not between sensory modalities. Nature 387, 808–810 (1997). 9194561 10.1038/42947

[b13] Hein G., Parr A. & Duncan J. Within-modality and cross-modality attentional blinks in a simple discrimination task. Percept. Psychophys. 68, 54–61 (2006). 16617829 10.3758/bf03193655

[b14] Soto-Faraco S. & Spence C. Modality-specific auditory and visual temporal processing deficits. Q. J. Exp. Psychol. A 55, 23–40 (2002). 11873849 10.1080/02724980143000136

[b15] Stock A.-K., Arning L., Epplen J. T. & Beste C. DRD1 and DRD2 genotypes modulate processing modes of goal activation processes during action cascading. J. Neurosci. Off. J. Soc. Neurosci. 34, 5335–5341 (2014). 10.1523/JNEUROSCI.5140-13.2014 PMC6608997 24719111

[b16] Cosmelli D. *et al.* Shifting visual attention away from fixation is specifically associated with alpha band activity over ipsilateral parietal regions. Psychophysiology 48, 312–322 (2011). 20663090 10.1111/j.1469-8986.2010.01066.x

[b17] Talsma D., Senkowski D. & Woldorff M. G. Intermodal attention affects the processing of the temporal alignment of audiovisual stimuli. Exp. Brain Res. 198, 313–328 (2009). 19495733 10.1007/s00221-009-1858-6 PMC2733193

[b18] Otto T. U., Dassy B. & Mamassian P. Principles of multisensory behavior. J. Neurosci. Off. J. Soc. Neurosci. 33, 7463–7474 (2013). 10.1523/JNEUROSCI.4678-12.2013 PMC6619564 23616552

[b19] Koechlin E. & Summerfield C. An information theoretical approach to prefrontal executive function. Trends Cogn. Sci. 11, 229–235 (2007). 17475536 10.1016/j.tics.2007.04.005

[b20] Koechlin E., Ody C. & Kouneiher F. The architecture of cognitive control in the human prefrontal cortex. Science 302, 1181–1185 (2003). 14615530 10.1126/science.1088545

[b21] Jeon H.-A. & Friederici A. D. Two principles of organization in the prefrontal cortex are cognitive hierarchy and degree of automaticity. Nat. Commun. 4, 2041 (2013). 23787807 10.1038/ncomms3041

[b22] Paraskevopoulos E., Kuchenbuch A., Herholz S. C. & Pantev C. Musical expertise induces audiovisual integration of abstract congruency rules. J. Neurosci. Off. J. Soc. Neurosci. 32, 18196–18203 (2012). 10.1523/JNEUROSCI.1947-12.2012 PMC6621720 23238733

[b23] Kuchenbuch A., Paraskevopoulos E., Herholz S. C. & Pantev C. Audio-tactile integration and the influence of musical training. PloS One 9, e85743 (2014). 24465675 10.1371/journal.pone.0085743 PMC3897506

[b24] Beste C., Stock A.-K., Epplen J. T. & Arning L. On the relevance of the NPY2-receptor variation for modes of action cascading processes. NeuroImage 10.1016/j.neuroimage.2014.08.026 (2014). 25157429

[b25] Alais D., Morrone C. & Burr D. Separate attentional resources for vision and audition. Proc. Biol. Sci. 273, 1339–1345 (2006). 16777721 10.1098/rspb.2005.3420 PMC1560294

[b26] Spence S. A. *et al.* Behavioural and functional anatomical correlates of deception in humans. Neuroreport 12, 2849–2853 (2001). 11588589 10.1097/00001756-200109170-00019

[b27] Turatto M., Benso F., Galfano G. & Umiltà C. Nonspatial attentional shifts between audition and vision. J. Exp. Psychol. Hum. Percept. Perform. 28, 628–639 (2002). 12075893 10.1037//0096-1523.28.3.628

[b28] Brisson B., Robitaille N. & Jolicoeur P. Stimulus intensity affects the latency but not the amplitude of the N2pc. Neuroreport 18, 1627–1630 (2007). 17885614 10.1097/WNR.0b013e3282f0b559

[b29] Buzzell G., Chubb L., Safford A. S., Thompson J. C. & McDonald C. G. Speed of human biological form and motion processing. PloS One 8, e69396 (2013). 23894467 10.1371/journal.pone.0069396 PMC3722264

[b30] Luck S. J., Woodman G. F. & Vogel E. K. Event-related potential studies of attention. Trends Cogn. Sci. 4, 432–440 (2000). 11058821 10.1016/s1364-6613(00)01545-x

[b31] Hopf J.-M., Vogel E., Woodman G., Heinze H.-J. & Luck S. J. Localizing visual discrimination processes in time and space. J. Neurophysiol. 88, 2088–2095 (2002). 12364530 10.1152/jn.2002.88.4.2088

[b32] Luck S. J., Heinze H. J., Mangun G. R. & Hillyard S. A. Visual event-related potentials index focused attention within bilateral stimulus arrays. II. Functional dissociation of P1 and N1 components. Electroencephalogr. Clin. Neurophysiol. 75, 528–542 (1990). 1693897 10.1016/0013-4694(90)90139-b

[b33] Vogel E. K. & Luck S. J. The visual N1 component as an index of a discrimination process. Psychophysiology 37, 190–203 (2000). 10731769

[b34] Patel S. H. & Azzam P. N. Characterization of N200 and P300: selected studies of the Event-Related Potential. Int. J. Med. Sci. 2, 147–154 (2005). 16239953 10.7150/ijms.2.147 PMC1252727

[b35] Verleger R., Jaśkowski P. & Wascher E. Evidence for an integrative role of P3b in linking reaction to perception. J. Psychophysiol. 19, 165–181 (2005).

[b36] Botvinick M. M., Cohen J. D. & Carter C. S. Conflict monitoring and anterior cingulate cortex: an update. Trends Cogn. Sci. 8, 539–546 (2004). 15556023 10.1016/j.tics.2004.10.003

[b37] Rushworth M. F. S., Walton M. E., Kennerley S. W. & Bannerman D. M. Action sets and decisions in the medial frontal cortex. Trends Cogn. Sci. 8, 410–417 (2004). 15350242 10.1016/j.tics.2004.07.009

[b38] Botvinick M. M. Conflict monitoring and decision making: reconciling two perspectives on anterior cingulate function. Cogn. Affect. Behav. Neurosci. 7, 356–366 (2007). 18189009 10.3758/cabn.7.4.356

[b39] Bryson J. Cross-paradigm analysis of autonomous agent architecture. J. Exp. Theor. Artif. Intell. 12, 165–189 (2000).

[b40] Heyder K., Suchan B. & Daum I. Cortico-subcortical contributions to executive control. Acta Psychol. (Amst.) 115, 271–289 (2004). 14962404 10.1016/j.actpsy.2003.12.010

[b41] Prescott T. J., Bryson J. J. & Seth A. K. Introduction. Modelling natural action selection. Philos. Trans. R. Soc. Lond. B. Biol. Sci. 362, 1521–1529 (2007). 17428783 10.1098/rstb.2007.2050 PMC2042525

[b42] Logan G. D. & Gordon R. D. Executive control of visual attention in dual-task situations. Psychol. Rev. 108, 393–434 (2001). 11381835 10.1037/0033-295x.108.2.393

[b43] Rubinstein J. S., Meyer D. E. & Evans J. E. Executive control of cognitive processes in task switching. J. Exp. Psychol. Hum. Percept. Perform. 27, 763–797 (2001). 11518143 10.1037//0096-1523.27.4.763

[b44] Walsh N. D., Seal M. L., Williams S. C. R. & Mehta M. A. An investigation of cognitive ‘branching’ processes in major depression. BMC Psychiatry 9, 69 (2009). 19903326 10.1186/1471-244X-9-69 PMC2777899

[b45] Gajewski P. D. & Falkenstein M. Diversity of the P3 in the task-switching paradigm. Brain Res. 1411, 87–97 (2011). 21803343 10.1016/j.brainres.2011.07.010

[b46] Fisher A. V. Automatic shifts of attention in the Dimensional Change Card Sort task: subtle changes in task materials lead to flexible switching. J. Exp. Child Psychol. 108, 211–219 (2011). 20674930 10.1016/j.jecp.2010.07.001

[b47] Ramnani N. & Owen A. M. Anterior prefrontal cortex: insights into function from anatomy and neuroimaging. Nat. Rev. Neurosci. 5, 184–194 (2004). 14976518 10.1038/nrn1343

[b48] Koechlin E. & Hyafil A. Anterior prefrontal function and the limits of human decision-making. Science 318, 594–598 (2007). 17962551 10.1126/science.1142995

[b49] Koechlin E., Basso G., Pietrini P., Panzer S. & Grafman J. The role of the anterior prefrontal cortex in human cognition. Nature 399, 148–151 (1999). 10335843 10.1038/20178

[b50] Clerget E., Badets A., Duqué J. & Olivier E. Role of Broca's area in motor sequence programming: a cTBS study. Neuroreport 22, 965–969 (2011). 22089648 10.1097/WNR.0b013e32834d87cd

[b51] Fazio P. *et al.* Encoding of human action in Broca's area. Brain J. Neurol. 132, 1980–1988 (2009). 10.1093/brain/awp118 19443630

[b52] Koechlin E. & Jubault T. Broca's area and the hierarchical organization of human behavior. Neuron 50, 963–974 (2006). 16772176 10.1016/j.neuron.2006.05.017

[b53] Logan G. D. & Cowan W. B. On the ability to inhibit thought and action: A theory of an act of control. Psychol. Rev. 91, 295–327 (1984). 10.1037/a0035230 24490789

[b54] Perrin F., Pernier J., Bertrand O. & Echallier J. F. Spherical splines for scalp potential and current density mapping. Electroencephalogr. Clin. Neurophysiol. 72, 184–187 (1989). 2464490 10.1016/0013-4694(89)90180-6

[b55] Nunez P. L. & Pilgreen K. L. The spline-Laplacian in clinical neurophysiology: a method to improve EEG spatial resolution. J. Clin. Neurophysiol. Off. Publ. Am. Electroencephalogr. Soc. 8, 397–413 (1991). 1761706

[b56] Pascual-Marqui R. D. Standardized low-resolution brain electromagnetic tomography (sLORETA): technical details. Methods Find. Exp. Clin. Pharmacol. 24 **Suppl D**, 5–12 (2002). 12575463

[b57] Pascual-Marqui R. D., Esslen M., Kochi K. & Lehmann D. Functional imaging with low-resolution brain electromagnetic tomography (LORETA): a review. Methods Find. Exp. Clin. Pharmacol. 24 **Suppl C**, 91–95 (2002). 12575492

[b58] Marco-Pallarés J., Grau C. & Ruffini G. Combined ICA-LORETA analysis of mismatch negativity. NeuroImage 25, 471–477 (2005). 15784426 10.1016/j.neuroimage.2004.11.028

[b59] Sekihara K., Sahani M. & Nagarajan S. S. Localization bias and spatial resolution of adaptive and non-adaptive spatial filters for MEG source reconstruction. NeuroImage 25, 1056–1067 (2005). 15850724 10.1016/j.neuroimage.2004.11.051 PMC4060617

[b60] Vitacco D., Brandeis D., Pascual-Marqui R. & Martin E. Correspondence of event-related potential tomography and functional magnetic resonance imaging during language processing. Hum. Brain Mapp. 17, 4–12 (2002). 12203683 10.1002/hbm.10038 PMC6872086

[b61] Fuchs M., Kastner J., Wagner M., Hawes S. & Ebersole J. S. A standardized boundary element method volume conductor model. Clin. Neurophysiol. Off. J. Int. Fed. Clin. Neurophysiol. 113, 702–712 (2002). 10.1016/s1388-2457(02)00030-5 11976050

